# Empowering Women Through Pharmaceutical Education: A New Approach to Premenstrual Syndrome

**DOI:** 10.3390/healthcare14030348

**Published:** 2026-01-30

**Authors:** Celia Piquer-Martínez, Manuel Gómez-Guzmán, Adriana González-Salgado, María Isabel Valverde-Merino, Francisco Javier Ferreira-Alfaya, Blete Isufi, Victoria García-Cárdenas, Francisco Rivas-García, María José Zarzuelo

**Affiliations:** 1 Department of Pharmacy and Pharmaceutical Technology, School of Pharmacy, University of Granada, 18071 Granada, Spain; celiapiquermartinez@gmail.com (C.P.-M.); adrianagonzalezqf@gmail.com (A.G.-S.); misabelvalverdemerino@gmail.com (M.I.V.-M.); ferre1605@gmail.com (F.J.F.-A.); bleteisufi@correo.ugr.es (B.I.); vcardenas@ugr.es (V.G.-C.); mjzarzuelo@ugr.es (M.J.Z.); 2 Department of Pharmacology, School of Pharmacy, University of Granada, 18071 Granada, Spain; mgguzman@ugr.es; 3 Instituto de Investigación Biosanitaria de Granada (ibs.GRANADA), 18012 Granada, Spain; 4 Municipal Health and Consumer Unit, Guadix City Council, 18500 Guadix, Spain

**Keywords:** premenstrual syndrome, quality of life, pharmacy, education

## Abstract

**Objective**: To evaluate the effectiveness of a pharmacist-led educational intervention in reducing premenstrual syndrome (PMS) symptoms and improving self-care practices. **Methods**: A mixed-design study was conducted in community pharmacies in Spain between January and June 2025. First, a cross-sectional analysis determined PMS prevalence. Second, a longitudinal pre-post study was performed with women suffering from PMS. The intervention involved personalized guidance and an evidence-based educational infographic. Primary outcomes included symptom severity (measured by a numeric rating scale) and quality of life. **Results**: 350 women participated in the study. The mean age of participants was 23.7 ± 6.3 years (range: 17–51 years). At the six-month follow-up, the PMS group showed a significant reduction in mean pain intensity (from 6.86 to 3.26; *p* < 0.001) and a smaller reduction in the control group (from 4.82 to 2.88; *p* < 0.001), alongside improvements in irritability, insomnia, and fatigue. The proportion of women reporting a negative impact on quality of life decreased from 97.0% to 60.8% (*p* < 0.001). Oral contraceptive use was identified as a protective factor (OR: 0.33; 95% CI: 0.17–0.65). **Conclusions**: Educational interventions led by community pharmacists are effective in significantly alleviating PMS symptoms and enhancing women’s quality of life. Practice Implications: Community pharmacists are strategically positioned to identify women with PMS and provide evidence-based education. Implementing structured protocols and visual tools in pharmacies can optimize symptom management and promote self-care.

## 1. Introduction

Premenstrual syndrome (PMS) represents a complex convergence of physical, emotional, and behavioral symptoms that recur during the luteal phase of the menstrual cycle and remit with menstruation [1]. The etiology of PMS is multifactorial and not yet fully understood, though it is widely believed to result from an abnormal sensitivity to normal hormonal fluctuations—specifically estrogen and progesterone—during the luteal phase [1]. These hormonal changes interact with central neurotransmitters, such as serotonin and gamma-aminobutyric acid (GABA), influencing mood and pain perception [2]. Several risk factors have been correlated with the severity of PMS, including genetic predisposition, age, high body mass index (BMI), parity, and lifestyle factors such as smoking or high caffeine intake [1,2]. Furthermore, comorbidities like endometriosis or dysmenorrhea can exacerbate the clinical presentation. Symptoms are diverse, ranging from somatic complaints (breast tenderness, bloating, headache) to affective symptoms (irritability, anxiety, depression), which often have a more debilitating effect on the patient’s quality of life [3]. Although up to 90% of women of reproductive age report experiencing some premenstrual symptoms, it is estimated that 47.8% suffer from PMS, and between 24 and 32% experience moderate-to-severe symptoms that interfere with their daily lives [4]. Despite this high prevalence, PMS remains underdiagnosed and undertreated, often dismissed as an inevitable part of female physiology rather than a manageable condition [5].

To date, there have been no objective biochemical markers for PMS. Diagnosis relies on detailed anamnesis and prospective daily symptom tracking over at least two menstrual cycles. The hallmark is symptom onset restricted to the luteal phase, with complete remission during the follicular phase. Physical examination is essential to exclude differential diagnoses, and psychiatric evaluation may be warranted [6].

Current management strategies include lifestyle modifications—such as diet, exercise, and stress management—as first-line approaches, followed by pharmacological treatments like Selective Serotonin Reuptake Inhibitors (SSRIs) or hormonal contraceptives for more severe cases [7,8,9,10,11,12,13]. However, many women rely on self-care strategies without professional guidance, potentially leading to suboptimal symptom relief. While the benefits of health education are well-documented, few studies have specifically explored the role of community pharmacists as primary educators in menstrual health [14,15,16].

Community pharmacies are strategically positioned to bridge this gap due to their accessibility and the frequency with which women visit them for minor ailments or pain relief. However, there is a lack of structured, evidence-based pharmaceutical intervention protocols specifically designed to empower women with PMS through visual and educational tools.

The primary objective of this study was to evaluate the effectiveness of a structured, pharmacist-led educational intervention—supported by evidence-based infographics—in reducing the severity of PMS symptoms and improving self-care practices and quality of life among women in Spain. This study seeks to demonstrate that the community pharmacist can play a pivotal clinical role in the multidisciplinary management of women’s health.

## 2. Materials and Methods

### 2.1. Study Design

A mixed-methods study design was employed. Phase 1 (Cross-sectional): A descriptive study was conducted to determine the prevalence of PMS and compare characteristics between women with PMS (PMS group) and those without (control group). The control group was utilized solely for this baseline characterization and risk factor analysis. Phase 2 (longitudinal intervention): Women identified with PMS were invited to participate in a 6-month prospective intervention study (January to June 2025). A pre-post design was used to evaluate the effectiveness of the pharmaceutical intervention within the PMS group.

The intervention was delivered by community pharmacists who received prior training on the study protocol. The intervention consisted of the following:

1. Initial Counseling (Month 0): A standardized 15 min face-to-face session where the pharmacist explained the physiology of PMS and provided personalized lifestyle advice.
2. Educational Material: Participants received a digital infographic covering four key pillars: dietary changes (reducing caffeine/salt), regular aerobic exercise, stress management techniques, and evidence-based supplementation.
3. Follow-up (Months 1–5): To ensure fidelity and adherence, participants received monthly reinforcement videos (approx. 2 min each) via digital messaging, reiterating the key messages of the infographic.

### 2.2. Participants

Eligible participants were women experiencing PMS with no comorbid menstrual disorders.

The study population was calculated based on women with PMS in Spain. The sample size was calculated using the following formula:

n = N × Z^2^ × p (1 − p)/(N − 1) + e^2^ + Z^2^ × (1 − p)

Regarding sample size, the initial calculation (n = 373) was based on estimating prevalence with a 95% confidence interval. For the intervention phase, the final sample of n = 165 provided >80% power to detect a medium effect size (Cohen’s d = 0.5) in the reduction in pain scores.

#### 2.2.1. Inclusion Criteria

- Currently menstruating.
- Participants over 18 years of age.
- Inclusion in the PMS group was based on retrospective reporting of symptoms consistent with the American College of Obstetricians and Gynecologists (ACOG) criteria. Participants were screened for the presence of at least one affective and one somatic symptom during the five days prior to menses for three consecutive cycles. It is important to note that this screening constitutes a “PMS-like symptom” assessment rather than a prospective clinical diagnosis confirmed by daily charting over two months.

#### 2.2.2. Exclusion Criteria

- Pregnancy or breastfeeding.
- Menopause.

Participants were classified into a PMS group and a control group based on the presence or absence of PMS symptoms.

### 2.3. Questionnaire Design

The questionnaire was adapted from the validated tool developed by Sima et al. [17], translated into Spanish, and administered at the pharmacy. It consisted of four main sections:

1. Sociodemographic data: age, weight, height, city of residence, marital status, education level, employment status, and number of pregnancies.
2. Menstrual history: age at menarche, cycle regularity and duration, duration of menstrual bleeding, and presence of heavy flow.
3. PMS-associated factors: previous PMS diagnosis, autoimmune disease, endometriosis, polycystic ovary syndrome, infertility, sexual activity, use of oral contraceptives, physical activity level, regular healthy diet, tobacco, alcohol, and caffeine consumption, sleep duration, frequency and severity of PMS symptoms, and number of symptomatic days.
4. Pain and symptom management: intensity of menstrual pain, use of medications (non-steroidal anti-inflammatory drugs (NSAIDs), SSRIs, others), non-pharmacological methods (e.g., exercise, massage, relaxation, heat application), and use of nutritional supplements (e.g., evening primrose oil, vitamin B6, magnesium, iron).

Pain was assessed using a 1–10 scale (1 being best to 10 worst), both before and during PMS. Participants were also asked about the impact of symptoms on quality of life.

To assess the impact on quality of life (QoL), we utilized a specific item derived from the validated questionnaire by Sima et al. [17]. Participants were asked to evaluate whether their premenstrual symptoms interfered with their daily activities (including work, academic performance, and social interactions) using a self-perceived dichotomous scale (Yes/No). This measure, combined with the pain intensity score, provided a functional assessment of symptom severity.

In the 6-month follow-up questionnaire, sociodemographic items were omitted. Remaining questions were repeated, and new ones were added to assess which recommendations were implemented, whether any improvement was perceived, if participants sought medical advice, and if new pharmacological treatments were started.

### 2.4. Intervention

**Figure 1:**
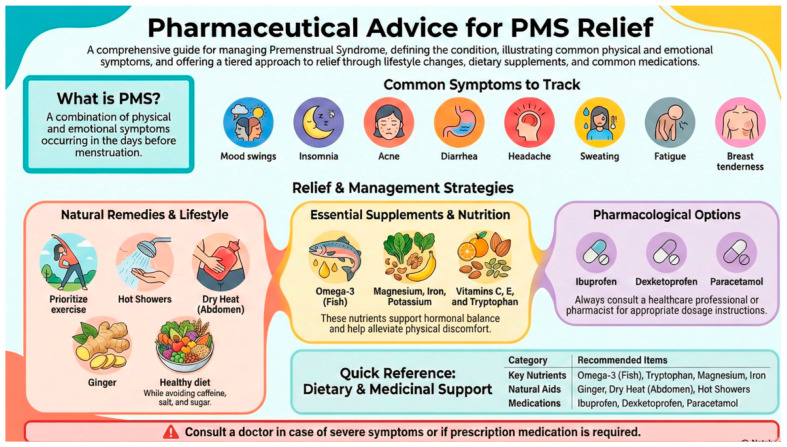
Digital infographic.

The intervention consisted of a structured pharmaceutical education program. Participants received a digital infographic via WhatsApp or email containing evidence-based recommendations on diet, exercise, and supplements (Figure 1). Data were collected at baseline and at a six-month follow-up to assess the impact of the intervention. It included the following PMS management recommendations:

- Non-pharmacological strategies: abdominal heat application, physical exercise, relaxation techniques, increased fluid intake, avoidance of sugar, salt, carbonated drinks, alcohol, and caffeine, and consumption of balanced, frequent, and moderate meals.
- Nutritional supplementation: recommended nutrients included ginger, evening primrose oil, vitamin B6 (from supplements or foods like bananas, nuts, green leafy vegetables, whole grains), vitamin C (e.g., kiwis, oranges, Brussels sprouts, citrus fruits, peppers), vitamin E (e.g., olive oil, leafy greens, avocado), omega-3 (e.g., fatty fish, walnuts), magnesium, iron, potassium, and tryptophan (from dairy, eggs, meat, fish, legumes, and nuts).
- Pharmacological recommendations: use of NSAIDs like ibuprofen or paracetamol in cases of moderate-to-severe pain. Participants with persistent or severe symptoms were advised to consult a healthcare provider.

Additionally, to reinforce adherence, six short videos (one per month) were sent to participants, covering specific topics such as diet, stress management, and exercise.

### 2.5. Statistical Analysis

Data analysis was performed using SPSS version 29 (SPSS Inc., Chicago, IL, USA). Continuous variables are presented as mean ± standard deviation (SD), and categorical variables are expressed as frequencies and percentages. For the cross-sectional phase, comparisons between the PMS group and the Control group were performed using Student’s *t*-test for independent samples (continuous data) and Chi-square tests (categorical data). Multivariate logistic regression analysis was performed to identify potential protective factors. The dependent variable was defined as the presence of PMS (PMS group = 1 vs. control group = 0), while supplement intake (tryptophan, iron, magnesium, vitamin B6, calcium) was included as independent binary variables (Yes/No). Results are expressed as odds ratios (ORs) with 95% confidence intervals (CIs). For the longitudinal intervention phase (within-subject analysis), changes in symptom severity (pain scores) were analyzed using paired *t*-tests. Changes in categorical variables (e.g., impact on quality of life) were analyzed using McNemar’s test. A *p*-value < 0.05 was considered statistically significant.

### 2.6. AI Statement

The image in the figure was created using Canva, a website (https://www.canva.com/es_es/) (accessed on 27 January 2026) that provides design tools.

## 3. Results

### 3.1. Sociodemographic and Clinical Profiles

A comprehensive demographic profile was obtained for all participants, recording variables such as age, civil status, educational level (primary, secondary, or university), and current occupation. These baseline characteristics are detailed in Table 1, allowing for a clear comparison between the control group and the PMS group.

The prevalence of PMS in the initial screening was 47.14%. A total of 350 women participated in the study, with 185 in the control group (without premenstrual syndrome, PMS) and 165 in the PMS group.

The mean age was 23.54 ± 6.02 years in the control group and 23.92 ± 6.63 years in the PMS group. BMI and age at menarche were similar between. However, significant differences were observed in pain scores both before PMS (2.42 ± 1.50 vs. 3.26 ± 2.24; *p* < 0.001) and during PMS (4.88 ± 2.23 vs. 6.86 ± 1.98; *p* < 0.001) (Table 1).

The majority of participants lived in urban areas in southern Spain (81.2% control, 82.9% PMS), were single (89.2% vs. 93.9%), had a university-level education (82.7% vs. 86.7%), and were employed (55.3% vs. 53.1%) (Table 1).

Regarding menstrual characteristics, heavy menstrual flow and a prior history of PMS were reported in a higher percentage in the PMS group. Endometriosis was more prevalent among women with PMS (8.0% vs. 1.6%, *p* = 0.004), while no significant differences were found for autoimmune diseases or polycystic ovary syndrome (Table 2).

The use of oral contraceptives was significantly lower in the PMS group (11.5% vs. 18.5%, *p* = 0.048). Analgesic use was significantly higher among women with PMS (NSAIDs or paracetamol: 71.2% vs. 63.5%, *p* = 0.002). A significant quality-of-life impact was reported by 97.0% of the PMS group and only 19.0% of the control group (Table 2).

### 3.2. Impact of the Educational Intervention

Healthy habits, including physical activity, diet, tobacco, alcohol, and coffee consumption, as well as sleep, were generally similar between groups. However, there was a borderline-significant trend toward greater coffee consumption in the PMS group (67.1% vs. 58.2%, *p* = 0.054). These lifestyle habits remained stable after the intervention.

The intervention resulted in a clinically relevant reduction in severe pain intensity, decreasing from a mean of 43.3% of women with PMS at baseline to 25.5% post-intervention (*p* < 0.001). Quality-of-life impairment was also significantly reduced post-intervention (from 97.0% to 60.8%, *p* < 0.001) (Table 3).

Detailed changes in specific symptoms (irritability, breast pain, swelling) are presented in Table 4. A significant improvement was observed across physical and psychological domains, including insomnia (27.9% to 17.6%, *p* = 0.026), nausea (28.5% to 19.6%, *p* = 0.002), dizziness (30.9% to 21.6%, *p* = 0.023), irritability (71.5% to 51.0%, *p* = 0.006) and fatigue (61.2% to 41.2%, *p* < 0.001) (Table 4).

### 3.3. Quality of Life and Associated Factors

The proportion of women reporting a negative impact on quality of life dropped from 97.0% to 60.8%. Multivariate analysis indicated that oral contraceptive use was a significant protective factor against moderate/severe PMS (OR = 0.329, 95% CI = 0.167–0.650) (Table 5).

Table 5 presents the logistic regression analysis regarding nutritional supplementation. A significant inverse association was observed between the intake of specific supplements and the presence of PMS. Specifically, women who reported consuming tryptophan (OR = 0.149, 95% CI = 0.070–0.318), iron (OR = 0.257, 95% CI = 0.101–0.656), vitamin B6 (OR = 0.217, 95% CI = 0.072–0.653), omega (OR = 0.229, 95% CI = 0.083–0.632), vitamin C (OR = 0.193, 95% CI = 0.092–0.406), vitamin E (OR = 0.242, 95% CI = 0.099–0.592), and magnesium (OR = 0.301, 95% CI = 0.114–0.794) showed lower odds of belonging to the PMS group compared to the control group. In addition, as expected, the likelihood that PMS symptomatology would impact quality of life was almost 3 times higher (OR = 2.892, 95% CI = 1.565–5.345) (Table 5).

A greater probability of presenting PMS was observed in those women who had a family history of PMS (OR = 2.615, 95% CI = 1.696–4.031), those with endometriosis (OR = 5.264, 95% CI = 1.472–18.819) and those who did not use oral contraceptives (OR = 1.605, 95% CI = 1.000–2.701). Women who had certain symptoms before menstruation were also associated with an increased likelihood of PMS: diarrhea (OR = 2.171, 95% CI = 1.415–3.333), arthralgia (OR = 2.692, 95% CI = 1.275–5.685), headache (OR = 3.003, 95% CI = 1.942–4.642), insomnia (OR = 3.820, 95% CI = 2.088–6.987), hyperhidrosis (OR = 4.314, 95% CI = 2.167–8.585), polyuria (OR = 3.095, 95% CI = 1.483–6.459), nausea (OR = 6.300, 95% CI = 3.139–12.648), vomiting (OR = 6.962, 95% CI = 2.346–20.658), loss of appetite (OR = 5.753, 95% CI = 2.990–11.066), dizziness (OR = 4.725, 95% CI = 2.568–8.695), agitation or irritability (OR = 5.783, 95% CI = 3.647–9.172), fatigue (OR = 3.635, 95% CI = 2.334–5.663) and general pain (OR = 0.443, 95% CI = 0.277–0.709) (Table 6).

## 4. Discussion

The present study demonstrates that a community pharmacist-led educational intervention significantly reduces the intensity of PMS symptoms and improves women’s quality of life.

Our findings reveal a drastic reduction in pain intensity (from 6.86 to 3.26, *p* < 0.001) and a decrease in the proportion of women reporting a negative impact on quality of life from 97.0% to 60.8% after six months.

The success of the intervention suggests that lack of knowledge regarding lifestyle and self-care is a major barrier to PMS management [18,19]. By providing evidence-based visual tools (infographics) and personalized counseling, pharmacists empowered women to adopt effective strategies such as local heat application, exercise, and targeted supplementation (vitamin B6, magnesium, omega-3). This aligns with the “gatekeeper” role of the pharmacist, moving beyond simple medication dispensing to proactive health education [20,21]. The significant reduction in psychological symptoms like irritability and insomnia highlights that somatic and affective symptoms are interconnected and responsive to lifestyle changes. Significant associations were found between specific strategies (e.g., vitamin E or a healthy diet) and the reduction in particular symptoms such as headache, diarrhea, and agitation, corroborating recent findings in young female populations [22]. As this was a non-randomized comparison, we cannot establish a direct causal relationship. The observed association might be influenced by the ‘healthy user bias,’ where women who take supplements may also systematically engage in other healthy behaviors (better diet, more exercise) that mitigate PMS symptoms. Therefore, while the association is promising, it does not confirm that supplements alone prevent PMS without controlled clinical trials.

Our results corroborate previous research indicating the benefits of non-pharmacological interventions. For instance, Ozeki et al. found that educational checklists improved menstrual symptoms and work productivity [23]. However, unlike general educational campaigns, our study highlights the added value of the community pharmacist’s active involvement, a gap identified by Suaidi et al. [5]. Regarding risk factors, our multivariate analysis confirmed that family history and endometriosis significantly increase PMS risk, consistent with findings by Katib et al. [24]. Furthermore, the protective effect of oral contraceptives observed in our study (OR = 0.33) reinforces clinical trial data suggesting hormonal stability mitigates symptoms [25].

Our findings align with previous research suggesting that health education significantly mitigates PMS symptoms. The reduction in somatic and psychological symptoms observed in our study is consistent with results reported by Sima et al. [17] and others utilizing educational models. The mechanism behind this improvement is likely rooted in increased health literacy and self-efficacy. By understanding the physiological basis of their symptoms and receiving actionable, evidence-based advice (e.g., reducing caffeine to lower irritability, using heat for pain), patients regain a sense of control. This empowerment promotes adherence to lifestyle modifications, which are the first-line defense against PMS inflammation and hormonal sensitivity.

These findings have direct implications for pharmacy practice. Implementing structured PMS protocols in community pharmacies can reduce the burden on primary care physicians and provide immediate relief for women. The use of accessible visual tools (infographics) proved to be an effective method for enhancing adherence to healthy habits [26].

This study has several limitations that must be considered. First, the study design for the intervention phase was a single-arm pre-post study without a concurrent control group. Consequently, we cannot rule out the influence of placebo effects or natural variations in symptoms over time on the observed improvements. Second, the diagnosis of PMS was based on retrospective self-reports rather than prospective daily symptom charting, which is the gold standard; this may introduce recall bias. Third, the sample characteristics (predominantly young, university-educated women) limit the generalizability of our findings to older women, those with lower socioeconomic status, or rural populations. Finally, while we monitored adherence to the educational advice, medication changes (e.g., starting hormonal contraceptives or analgesics) during the 6-month period were not controlled for in the statistical analysis, which could act as a confounding factor.

## 5. Conclusions

This study confirms that educational interventions led by community pharmacists are effective in significantly alleviating PMS symptoms—specifically pain, irritability, and fatigue—and enhancing women’s quality of life. The community pharmacy serves as a vital, underutilized health hub for detecting and managing menstrual disorders. Future health policies should consider integrating structured pharmaceutical education programs to promote women’s self-care and autonomy.

## Figures and Tables

**Table 1:** Sociodemographic data.

	Control		PMS		
	Mean	SD	Mean	SD	*p*-Value
Age (Years)	23.54	6.02	23.92	6.36	0.570
BMI (kg/m^2)^	22.55	3.30	22.75	3.39	0.570
Age at first menstruation (years)	12.08	1.84	12.22	1.55	0.490
Days of PMS	3.85	3.39	5.81	5.60	0.163
Pain before PMS (1–10)	2.42	1.50	3.26	2.24	<0.001 *
Pain during PMS (1–10)	4.88	2.23	6.86	1.98	<0.001 *
	**Control**		**PMS**		
	**N**	**%**	**N**	**%**	***p*** **-value**
Place of residence					
South urban	147	81.2	136	82.9	0.626
North urban	13	7.2	10	6.1	
Rural	7	3.9	5	3	
Coast	13	7.2	10	6.1	
Morocco	1	0.6	0	0	
Italy	1	0.6	2	1.2	
Marital status					
Single	165	89.2	155	93.9	0.183
Married	19	10.3	10	6.1	
Widow	1	0.5	0	0.0	
Level of education					
No studies	4	2.2	4	2.4	0.500
Bachelor	28	15.1	18	10.9	
University	153	82.7	143	86.7	
Occupation					
Unemployment	76	44.7	68	46.9	0.391
Active	94	55.3	77	53.1	

SD: standard deviation. PMS: premenstrual syndrome. BMI: body mass index. * *p* < 0.050, Student’s *t*-test and Chi−square test.

**Table 2:** Characteristics of menstruation in women with and without premenstrual syndrome.

	Control		PMS		
	N	%	N	%	*p*-Value
Heavy discharge					
No	25	47.2	13	34.2	0.154
Yes	28	52.8	25	65.8	
History of PMS					
No	118	63.8	66	40.2	<0.001 *
Yes	67	36.2	98	59.8	
Autoimmune disease					
No	175	94.6	147	89.6	0.063
Yes	10	5.4	17	10.4	
Endometriosis					
No	181	98.4	149	92.0	0.004 *
Yes	3	1.6	13	8.0	
Polycystic ovary					
No	163	88.6	137	84.6	0.174
Yes	21	11.4	25	15.4	
Active sex life					
No	75	40.5	60	36.4	0.245
Yes	110	59.5	105	63.6	
Oral contraceptives					
No	150	81.5	146	88.5	0.048 *
Yes	34	18.5	19	11.5	
Pain treatment					
None	17	10.0	7	4.3	0.002 *
NSAIDs or paracetamol	108	63.5	116	71.2	
Metamizole	45	26.5	39	23.9	

NSAID: nonsteroidal anti-inflammatory drug. PMS: premenstrual syndrome. * *p* < 0.050 Chi-square test.

**Table 3:** Characteristics and healthy habits related to premenstrual syndrome between the control and PMS groups at baseline and at the end of the study.

	Control		PMS Basal		PMS Month 6		
	n	%	N	%	N	%	*p*-Value
Duration of cycles							
Irregular	31	16.8	31	18.8	32	19.6	0.360
Regular	154	83.2	134	81.2	133	80.4	
Menstrual bleeding							
1–3 days	31	16.8	21	12.7	16	9.8	0.120
3–5 days	111	60.0	90	54.5	84	51.0	
>5 days	43	23.2	54	32.7	65	39.2	
Physical activity							
No physical activity	36	19.5	43	26.2	39	23.5	0.231
Regular	94	50.8	72	43.9	78	47.1	0.812
Active	55	29.7	49	29.9	48	29.4	
Regular healthy eating							
No	36	19.5	35	21.2	36	21.6	0.392
Yes	149	80.5	130	78.8	129	78.4	0.957
Smoking habit							
No	164	89.1	139	84.8	133	80.4	0.146
Yes	20	10.9	25	15.2	32	19.6	0.957
Alcohol							
No	168	90.8	140	84.8	142	86.3	0.061
Yes	17	9.2	25	15.2	23	13.7	0.803
Coffee							
No	77	41.8	54	32.9	52	31.4	0.054
Yes	107	58.2	110	67.1	113	68.6	0.796
Sleeping hours							
<6 h	25	13.5	17	10.3	26	15.7	0.984
6–9 h	153	82.7	144	87.3	129	78.4	
>9 h	7	3.8	4	2.4	10	5.9	
Intensity of pain							
Low	72	39.3	16	9.8	39	23.5	<0.001 *
Moderate	92	50.3	77	47.0	84	51.0	<0.001 *
Severe	19	10.4	71	43.3	42	25.5	
Impact on quality of life							
No	149	81.0	5	3.0	65	39.2	<0.001 *
Yes	35	19.0	160	97.0	100	60.8	

* *p* < 0.050 Chi-square test.

**Table 4:** Differences between symptoms of premenstrual syndrome.

	Control		PMS Basal		PMS Month 6			
	N	%	N	%	N	%	*p*-Value	*p*-Value
**Diarrhea**							**C vs. PMS**	**0 vs. 6**
No	116	62.7	72	43.6	62	37.3	<0.001 *	0.422
Yes	69	37.3	93	56.4	103	62.7		
**Arthralgia**								
No	174	94.1	141	85.5	145	88.2	<0.001 *	0.618
Yes	11	5.9	24	14.5	20	11.8		
**Headache**								
No	118	63.8	61	37.0	71	43.1	<0.001 *	0.431
Yes	67	36.2	104	63.0	94	56.9		
**Insomnia**								
No	168	90.8	119	72.1	136	82.4	<0.001 *	0.026 *
Yes	17	9.2	46	27.9	29	17.6		
**Hyperhidrosis**								
No	173	93.5	127	77.0	123	74.5	<0.001 *	0.719
Yes	12	6.5	38	23.0	42	25.5		
**Polyuria**								
No	174	94.1	138	83.6	145	88.2	<0.001 *	0.459
Yes	11	5.9	27	16.4	20	11.8		
**Nausea**								
No	174	94.1	118	71.5	133	80.4	<0.001 *	0.002 *
Yes	11	5.9	47	28.5	32	19.6		
**Vomits**								
No	181	97.8	143	86.7	145	88.2	<0.001 *	0.772
Yes	4	2.2	22	13.3	20	11.8		
**Loss of appetite**								
No	172	93.0	115	69.7	97	58.8	<0.001 *	0.002 *
Yes	13	7.0	50	30.3	68	41.2		
**Dizziness**								
No	169	91.4	114	69.1	130	78.4	<0.001 *	0.023 *
Yes	16	8.6	51	30.9	35	21.6		
**Agitation**								
No	129	69.7	47	28.5	81	49.0	<0.001 *	0.006 *
Yes	56	30.3	118	71.5	84	51.0		
**Fatigue**								
No	129	69.7	64	38.8	97	58.8	<0.001 *	<0.001 *
Yes	56	30.3	101	61.2	68	41.2		
**General Pain**								
No	112	60.5	128	77.6	120	72.5	<0.001 *	0.462
Yes	73	39.5	37	22.4	45	27.5		

* *p* < 0.050 Chi-square test.

**Table 5:** Relationship between the presence of premenstrual syndrome symptoms and habits and supplementation to combat them.

PMS Symptoms	OR	95% CI		*p*-Value
Active sex life	0.791	0.428	1.462	0.278
Oral contraceptives	0.329	0.167	0.650	0.002 *
Healthy eating	1.339	0.673	2.666	0.254
Smoking	0.674	0.304	1.497	0.221
Alcohol consumption	1.080	0.431	2.707	0.542
Coffee consumption	0.897	0.488	1.648	0.425
Applying heat	1.574	0.874	2.832	0.085
Massage	0.320	0.168	0.609	<0.001 *
Exercise	1.657	0.648	4.240	0.205
Eating small, frequent meals	2.220	0.222	22.203	0.443
Avoiding salt and sugar	2.220	0.222	22.203	0.220
Avoiding gas and coffee	1.739	0.493	6.129	0.290
Tryptophan	0.149	0.070	0.318	<0.001 *
Iron	0.257	0.101	0.656	0.007 *
Vitamin B6	0.217	0.072	0.653	0.010 *
Ginger	0.890	0.102	7.777	0.630
Omega	0.229	0.083	0.632	0.007 *
Vitamin C	0.193	0.092	0.406	<0.001 *
Vitamin E	0.242	0.099	0.592	0.003 *
Magnesium	0.301	0.114	0.794	0.020 *
Quality of life	2.892	1.565	5.345	<0.001 *

PMS: premenstrual syndrome. OR: odds ratio. 95% CI: 95% confidence interval. * *p* < 0.050 Chi-square test.

**Table 6:** The relationship of premenstrual syndrome with its characteristics and symptoms.

PMS	OR	95% CI		*p*-Value
History of PMS	2.615	1.696	4.031	<0.001 *
Endometriosis	5.264	1.472	18.819	0.004 *
No Oral Contraceptives	1.605	1.000	2.701	0.048 *
Diarrhea	2.171	1.415	3.333	<0.001 *
Arthralgia	2.692	1.275	5.685	0.006 *
Headache	3.003	1.942	4.642	<0.001 *
Insomnia	3.820	2.088	6.987	<0.001 *
Hyperhidrosis	4.314	2.167	8.585	<0.001 *
Polyuria	3.095	1.483	6.459	0.001 *
Nausea	6.300	3.139	12.648	<0.001 *
Vomiting	6.962	2.346	20.658	<0.001 *
Loss of Appetite	5.753	2.990	11.066	<0.001 *
Dizziness	4.725	2.568	8.695	<0.001 *
Agitation	5.783	3.647	9.172	<0.001 *
Fatigue	3.635	2.334	5.663	<0.001 *
General Pain	0.443	0.277	0.709	<0.001 *

PMS: premenstrual syndrome. OR: odds ratio. 95% CI: 95% confidence interval. * *p* < 0.050 Chi-square test.

## Data Availability

The original contributions presented in this study are included in the article. Further inquiries can be directed to the corresponding author.

## Abbreviation

The following abbreviation is used in this manuscript:

PMS	Premenstrual Syndrome
